# Wavelet-based Gaussian-mixture hidden Markov model for the detection of multistage seizure dynamics: A proof-of-concept study

**DOI:** 10.1186/1475-925X-10-29

**Published:** 2011-04-19

**Authors:** Alan WL Chiu, Miron Derchansky, Marija Cotic, Peter L Carlen, Steuart O Turner, Berj L Bardakjian

**Affiliations:** 1 Biomedical Engineering Department, Louisiana Tech University, Ruston, Louisiana, USA; 2 Toronto Western Research Institute, University of Toronto, Toronto, Ontario, Canada; 3 Institute of Biomaterials and Biomedical Engineering, University of Toronto, Toronto, Ontario, Canada

## Abstract

**Background:**

Epilepsy is a common neurological disorder characterized by recurrent electrophysiological activities, known as seizures. Without the appropriate detection strategies, these seizure episodes can dramatically affect the quality of life for those afflicted. The rationale of this study is to develop an unsupervised algorithm for the detection of seizure states so that it may be implemented along with potential intervention strategies.

**Methods:**

Hidden Markov model (HMM) was developed to interpret the state transitions of the *in vitro *rat hippocampal slice local field potentials (LFPs) during seizure episodes. It can be used to estimate the probability of state transitions and the corresponding characteristics of each state. Wavelet features were clustered and used to differentiate the electrophysiological characteristics at each corresponding HMM states. Using unsupervised training method, the HMM and the clustering parameters were obtained simultaneously. The HMM states were then assigned to the electrophysiological data using expert guided technique. Minimum redundancy maximum relevance (mRMR) analysis and Akaike Information Criterion (AICc) were applied to reduce the effect of over-fitting. The sensitivity, specificity and optimality index of chronic seizure detection were compared for various HMM topologies. The ability of distinguishing early and late tonic firing patterns prior to chronic seizures were also evaluated.

**Results:**

Significant improvement in state detection performance was achieved when additional wavelet coefficient rates of change information were used as features. The final HMM topology obtained using mRMR and AICc was able to detect non-ictal (interictal), early and late tonic firing, chronic seizures and postictal activities. A mean sensitivity of 95.7%, mean specificity of 98.9% and optimality index of 0.995 in the detection of chronic seizures was achieved. The detection of early and late tonic firing was validated with experimental intracellular electrical recordings of seizures.

**Conclusions:**

The HMM implementation of a seizure dynamics detector is an improvement over existing approaches using visual detection and complexity measures. The subjectivity involved in partitioning the observed data prior to training can be eliminated. It can also decipher the probabilities of seizure state transitions using the magnitude and rate of change wavelet information of the LFPs.

## Background

Epilepsy is one of the most common neurological disorders, affecting over 50 million people worldwide. The disorder is characterized by spontaneous, recurrent, seemingly unpredictable symptoms called seizures [1,2]. A seizure can be defined as the sudden manifestation of lowered complexity synchronized rhythmic activities across populations of neurons [3]. Epilepsy affects motor and speech, as well as other cognitive functions that, if untreated, can lead to permanent damage to the brain. Time-frequency representation (TFR) is an important marker for understanding the progression into seizure onsets [4]. The progression to the seizure onset is associated with the entrainment of neuronal population firing. In particular, the TFR of epileptiform oscillation has been suggested as a useful tool in localizing regions of seizure onsets and in understanding the mechanisms behind seizure generation [5]. The current methods for detecting the state transitions of seizure episodes based on TFR usually involve the computation of state-specific features through supervised learning techniques [6-10]. In general, supervised learning strategies require certain knowledge of the system so that the data can be separated into different states based on their known dynamics before the training process. To date, a great deal of subjectivity is required for the implementation of these algorithms since the desired solution for detection must be defined by the experimenter.

Seizure detection refers to the identification of seizure onsets a few seconds before the observable behavioral changes [11,12]. Several effective supervised pattern recognition strategies have been developed for seizure detection. Multi-layered networks were first introduced to analyze EEG data pertaining to seizure phenomenon in the mid-1990s [13,14]. Various methods such as autoregressive models [15,16] to more advanced techniques such as support vector machines [17,18] were also proposed. The feature space for these approaches ranged from spectrogram [19], dominant frequency, power and amplitude [20] to time frequency distribution [21] such as wavelet transform based approximate entropy [22,23]. It has been reported that frequency content of neuronal electrical activity changed significantly during the progression of a seizure, both in the interspike interval and in the intraburst dynamics [8,24]. The major disadvantage of these supervised methods is that their performances can only be as good as the initial data separation criteria. Therefore, it is essential to utilize an unsupervised learning paradigm that transcends these restrictions.

An unsupervised probabilistic approach for the detection of seizure-like events (SLEs) *in vitro *extracellular local field potentials (LFPs) seizures using hidden Markov model (HMM) [25,26] along with clustering of wavelet features is proposed. The *in vitro *model, emulating human epilepsy, provides a platform for testing the seizure dynamics detection algorithm. The HMM is not meant to reproduce the exact electrophysiological recordings of the brain. Instead, it is used to capture the essential TFR characteristics in the progression of SLEs and to estimate the state transitions as a multi-stage process. The HMM has an advantage over the supervised approaches because it does not require prior manual separation of data into different dynamics. The current approach of detecting seizure events using Markov models involves the estimation of either two (seizure and interictal) [27] or three (baseline, detected and seizure) [28] distinct states. Even though these proposed methods appear to detect seizure onsets, they failed to address the possibility of having multiple distinctive dynamics between non-ictal (interictal) and chronic seizure events, which may be an important aspect for the development of seizure therapy techniques. The training process of the HMM is an unsupervised approach. However, a certain amount of bias can be achieved by making an informed choice on the assignment of the model states to the electrographical activities after the unsupervised training process is completed.

To determine the optimal HMM topology for seizure detection, two methods were proposed and compared. First, the performance of the trained HMM was evaluated on the validation set to determine the suitable HMM topology. Second, minimum redundancy maximum relevance (mRMR) analysis [29] and Akaike Information Criteria (AICc) [30,31] were used to find a suitable feature space and optimum model by balancing the log-likelihood (LL) against the number of model parameters. Furthermore, the performances of HMMs were compared with the wavelet-based supervised machine learning techniques [6,7] based on the sensitivity and specificity of chronic seizure detection, the detection delay and the optimality measure [32]. We hypothesized that using appropriate wavelet features, the HMM can detect the various stages of SLEs at least as well as, if not better than, the supervised machine learning algorithms. We also hypothesized that the optimal HMM topology can illuminate multiple transitional characteristics in the tonic firing phrase leading to the onsets of chronic seizure activities.

## Methods

### A. Tissue preparation and data acquisition

Hippocampal slice recordings were obtained from eight Wistar rats (17-25 days old). The animals were anaesthetized with halothane and decapitated in accordance with the Canadian Animal Care Guidelines. The brains were promptly dissected and maintained in ice-cold (4°C) artificial cerebrospinal fluid (aCSF) for four to five minutes. Each brain was incised in a horizontal manner in accordance with the procedure outlined by [33,34]. The dorsal cortex of each hemisphere was cut parallel to the rostral/caudal axis and glued dorsal side down to an aluminium block, with caudal end towards the blade. The block was secured at a 12-14° angle, and brain slices of 400 μm thick were sectioned using a vibratome. Next, slices were maintained at room temperature in oxygenated "standard" aCSF (95% O_2_, 5% CO_2_) for a minimum of one hour prior to recording. The composition of the "standard" aCSF was as follows (in mM), NaCl (125), KCl (5), NaH_2_PO_4 _(1.25), MgSO_4 _(2), CaCl_2 _(2), NaHCO_3 _(25) and glucose (10). The pH was approximately 7.4, with osmolarity in the range of 300 ± 5 mOsm. During the data acquisition stage, slices were transferred to the fusion chamber maintained at 30°C (Medical Systems Corp., Model PDMI-2, Harvard Apparatus, St. Laurent, Quebec, Canada). The pyramidal cells were visualized with an upright microscope (BX51, Olympus, Melville, NY, USA) using infrared imaging with differential interference contrast (IR-DIC) under 40 × magnification (water-immersion objective) with an OLY-150IR camera-video monitor unit (Olympus) [35]. Pyramidal cells were identified based on their characteristic spike frequency of 15.0 ± 5 Hz, their morphological features and the general location of the electrode placement. At the time of recording, spontaneous SLEs were induced by perfusing the slice with low-Mg^2+ ^ACSF (containing in mM: 123 NaCl, 5 KCl, 1.5 CaCl2, 0.25 MgSO4, 25 NaHCO3, 1.2 NaH2PO4 and 15 glucose), or by tetanic stimulation of the CA3 region in "standard" aCSF, once every 10 min (80 Hz, 1 second duration), using a Grass S44 stimulator (Grass Medical Instruments, West Warwick, Rhode Island). The reduction of extracellular Mg^2+ ^concentration has long been known to enhance neuronal excitability by decreasing membrane surface charge screening and, thereby, facilitating the activation of inward currents in addition to increasing the synaptic excitation by unblocking the NMDA receptor [36]. The population dynamic of the LFPs were measured using an aCSF-filled borosilicate glass pipette located in stratus pyramidal of the CA1 region of the hippocampus. Data was acquired using a custom-made DC differential amplifier with a lowpass filter (corner frequency 400 Hz), digitized at 1 kHz by a Digidata 1322 (Axon Instruments, Union City, California). The whole-cell patch-clamp recordings were performed in the current clamp configuration using an Axopatch 200B amplifier (Axon Instruments, Union City, CA, USA). The whole-cell patch pipette solutions contained (contained in mM: 8 NaCl, 0.001 CaCl_2_, 10 Na-Hepes, 5 KCl, 140 potassium gluconate, 1 MgCl_2_, 0.3 Na-GTP and 2 Na-ATP). The perforated patch pipette solution (containing in mM: 50 KCl, 2 Hepes, 0.1 EGTA and ≤50 μg/ml gramicidin).

Overall, this study utilized 50 SLEs from 20 hippocampal slices in 8 rats with at least two SLEs recordings per rat. Each SLE was normalized in amplitude between -1 and +1. The DC components and the 60 Hz noise along with their harmonics were removed using FIR notch filtering. All of the analyses were performed using MATLAB (MathWorks, Natick, MA). The entire date set contained SLEs 50-379 s in duration (mean ± standard deviation of 145 ± 70 s). A typical SLE was made up of the interictal period, followed by tonic firing and then chronic seizure, before returning to the interictal period through the postictal activity. The mean duration of the chronic seizure was 74 s with standard deviation of 32 s. The duration of the tonic firing pattern lasted 0-301 s (mean ± standard deviation of 49 ± 48 s). We also included over 30 min of non-ictal/interictal bursting (IB) activities to determine the false positive rates of the seizure detectors. Details of the dataset for analysis are given in Table 1.

**Table 1:** Characteristics of the data set.

Characteristics	
Duration of EEG recording	121 m
Number of seizures	50
Duration of non-ictal activity	30 m
Mean ± Stdev of tonic firing	49.8 ± 48.0 s
Mean ± Stdev of chronic seizure	73.6 ± 32.4 s
Sampling rate	1024 Hz

### B. Wavelet-based hidden Markov model seizure detector

The HMM is a powerful technique for the estimation and analysis of state transitions in any potentially multi-stage process. It is particularly useful in describing the progression of time-varying phenomenon in which the observed signals are emitted from the underlying dynamical states whose detailed generating mechanism is unknown or hidden. This is also important because the underlying dynamics of the brain is still relatively unknown.

#### Feature space

The LFPs recorded near the stratus pyramidal of the CA1 region of *in vitro *rat hippocampal slices were used to train the HMM. The continuous wavelet transform (CWT) [37-39] using Morlet mother wavelet *ψ(t) *was performed to extract the features. We have noted from our previous study that the choice of mother wavelet does not affect the pattern recognition system [6]. The modified wavelets *ψ*_*s,l*_(*t*), derived from a mother wavelet by a scaling factor *s *and a translation factor *l*, is defined as:

The wavelet coefficients (*c*) can be computed at each non-overlapping 1-second moving time window, as the correlation between the modified wavelet and the input data. For this study, seven frequency components (as illustrated in Table 2) denoting important physiologically-relevant frequency bands were obtained. We evaluated two feature spaces. The first feature set was made up of 7-D wavelet coefficient data, which was identical to the feature set used in the previous supervised seizure detection algorithm called the wavelet artificial neural networks (WANN) [6,7]. The second feature set was a 14-D vector consisted of the information from the first feature set and the rate of change information *Δc*, defined as the numerical difference of *c *from the previous time window at each frequency band.

**Table 2:** Feature space for the HMM.

Bands	Frequency Range (Hz)
Delta	<4
Theta	4 - 8
Alpha	8 - 15
Beta	15 - 40
Gamma	40 - 100
Super gamma	100 - 250
Fast ripple	250 - 400

The wavelet coefficients were divided into seven frequency bands according to the accepted physiological ranges.

#### HMM topology

The HMM topology is defined by the number of states (Q) in the HMM and the number of basis functions (M) used to represent the feature space. The value Q can be interpreted as the potential number of distinct dynamics that may exist within a SLE. The value M can loosely be interpreted as the complexity of the feature for each possible model state.

The model parameters of the HMM help create two probability functions: the state transition probability and the emission probability. The parameter *a*_*ij *_is the probability of state transition from state *i *to state *j *where *i *and *j *can range from 1 to Q. The model state *j *at time *t *is denoted as (*S*_*t *_= *j*).

In this study, Q could go up to 10, allowing for the detection of potential multi-stage SLE processes. The transition of the model states was also assumed to follow a static probability distribution such that the transition probability was independent of time.

**Figure 1:**
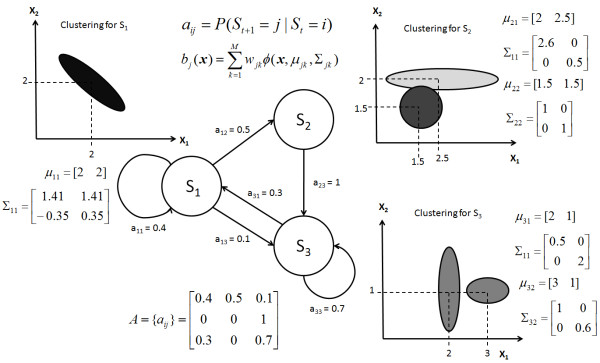
**General schematic representation of hidden Markov model**. A sample 3-state HMM with 2-D features space is illustrated. The state transition probably from state *i *to *j *is denoted by *a*_*ij *_and the emission probability of having feature ***x ***in state *j *is denoted as *b*_*j*_*(x)*. The mean *μ*_*jk *_and covariance matrix *Σ*_*jk *_of each cluster provide information on the center and distribution of information in the feature space.

For the representation of the features space, an unsupervised Mixture of Gaussians (MoGs) clustering technique was used. The feature vectors in the D-dimensional space is denoted as ***x***, where D = 7 or 14 depending of the features. The multivariate Gaussian probability density measure is a function of the center *μ*_*k *_(D by 1 in size) and covariance matrix *Σ*_*k *_(D by D in size). It is defined as:

The HMM was set up such that each state *j *would have M number of clusters in the feature space. Initially, the Gaussian clusters were randomly scattered and a subset of the feature ***x ***was assigned to the k^th ^cluster in the hidden state *j*. The output probability function, called emission probability *b*_*j*_*(****x****) *can then be defined as:

Here, the weighting factor for each cluster is denoted as *w*_*jk*_. In order to interpret *b*_*j*_*(****x****) *as true probability, the weighting factor needs to satisfy the following criteria:

In this paper, the number of clusters was allowed to vary from 1 to 5. A schematic representation of a 3-state HMM based on a 2-D feature space is shown in Figure 1, illustrating how the transition probabilities and the emission probabilities can tell us about the progression of the model states and the representation of Gaussian clusters in the state space.

#### Training and validation

The training of HMM involved the process of maximizing the probability of fitting the distribution of the feature space through an iterative process of updating the model parameters [40]. Forty percent of the SLEs were randomly selected as the training set. For each HMM topology (Q = 1 to 10 and M = 1 to 5), the model parameters (*a*_*ij*_*, w*_*jk*_*, μ*_*jk *_and *Σ*_*jk*_) related to HMM transitions and clustering were randomly initialized and estimated in an iterative manner simultaneously. These parameters were updated according to the Expectation-Maximization (EM) algorithm [25]. At the Expectation step, the responsibility of the model in representing the observed data was evaluated. The joint probability of observing all the data up to time *t *at state *j *was given as *α*_*j*_*(t) *and the conditional probability of all the future data from time *t+1 *onward at state *i *was given as *β*_*i*_*(t)*. The joint probability and conditional probability can be updated in an iterative manner:

Using α and β, the probability of *S*_*t *_= *i *is given by *γ*_*i*_*(t)*. The probability of having *S*_*t *_= *i *and *S*_*t+1 *_= *j *is denoted as *ζ*_*ij*_*(t)*.

For the clustering of feature space using MoGs, the probability that the k^th ^cluster at state *j *can generate a particular observation ***x***_***t ***_is given by:

From these estimates, the model parameters can be updated in the Maximization step.

The EM algorithm was repeated until the log-likelihood (LL) measure was no longer showing significant improvement. The LL measures quantified the goodness of fit between the feature ***x***:

The EM algorithm would typically take only a few seconds to complete. From the learning process described above, it should be obvious that if the number of clusters (M) and the number of possible HMM states (Q) were allowed to increase unrestricted, the LL would continue to improve at the expense of over-fitting the data.

Two methods were used to select the appropriate HMM model topology (Q and M) for each feature set. The first method involved the use of the validation data set. The performance of the trained HMMs at different Q and M combinations were examined on the validation sets consisting of 20% of the overall SLEs data. The HMM associated with the best overall balance between ictal and non-seizure data detection was denoted as the optimal HMMs. The optimal HMM obtained using 7-D wavelet coefficient features was denoted HMM_opt7D _and the optimal HMM obtained using 14-D wavelet and rate of change features was denoted HMM_opt14D_. These models were then tested to evaluate the state transitions on the test data, consisting of the remaining 40% of the SLEs. The second method to find the HMM topology involved the use of minimum redundancy maximum relevance (mRMR) analysis [41] to reducing the dimensionality of the feature space, and the use of AICc [30]. The mRMR method, which has been frequently used in gene expression research, can identify subsets of feature that were relevant to the classification tasks. It selected the features that were mutually far apart from each other (small *W*_*c*_) while still having a strong correlation (large *V*_*F*_) to the target state.

Here, *Π *represents the set of features, *C(f*_*m*_*,f*_*n*_*) *is the correlation between the two wavelet features *f*_*m *_and *f*_*n *_and *F(f*_*m*_*,S) *is the F-statistics between the feature *f*_*m *_and the target chronic seizure state *S*. The wavelet features associated with the largest mutual information quotient *V*_*F*_*/W*_*c *_were selected to construct the reduced feature set. This reduced feature set was then used to build other HMMs based on AICc. The AICc rewards goodness of fit based on the LL information, but also includes a penalty term that is proportional to the number of parameters (K = 3QM + Q^2^) to reduce over-fitting.

The size of the test set (*n*) also plays a role in determining AICc if *n/K < 40*. For each HMM topology (Q and M), an AICc value was computed. These AICc values were then rescaled with respect to the minimum AICc within the group such that the lowest AICc value was set to zero [31].

The model with ΔAICc < 0.25 while having the least number of model parameters (K) was then denoted as the optimal HMM (HMM_AIC_).

### C. Statistical test and optimality index

The HMM topologies were evaluated according to their abilities to detect non-seizure events, different stages in the tonic activities as well as chronic seizures. After the unsupervised training, the marginal posterior distribution *γ*_*i*_*(t) *for each state *i *was computed by evaluating the HMMs on the test data. The detection of distinct initiation and termination of seizure dynamics as they evolved with time [42] was also considered in the form of early and late tonic spikes. An expert-guided state assignment procedure was used to identify non-ictal to ictal transitions using short time maximum Lyapunov exponent estimator derived from Rosenstein's algorithm [43]. The dynamics of chronic seizure period was first assigned to the most probable HMM state after training. Based on the state progression in the transition matrix, the tonic firing and non-ictal states were assigned in reverse order to HMM states before chronic activities; the postictal activity was assigned to the appropriate state forward-in-time to the HMM state after chronic activities.

**Figure 2:**
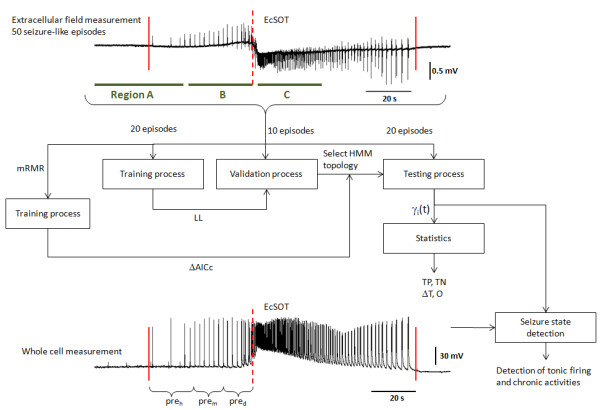
**Graphical representation on the method of evaluating HMMs**. On the top illustration, the LFP is illustrated. Maximum Lyapunov exponent analysis is used to determine the initiation and termination of seizure activities (marked by solid vertical lines). The seizure detection horizon is defined as a 1 min time window centered on the EcSOT (marked by dotted vertical line) and is denoted by regions B and C. After the initial training process (using 40% of the overall dataset) on the full 7-D and 14-D feature space, the performance of each HMM is evaluated using the validation set (20% of the overall dataset) to determine the HMM_opt7D _and HMM_opt14D _topology. Afterwards, feature reduction from mRMR analysis and AICc are used to find a suitable HMM_AIC _that balance the LL against the number of model parameters. The statistical tests (TP and TN) as well as optimality index (*O*) are then evaluated. On the bottom illustration, the intracellular activity before the EcSOT is partitioned based on its polarity characteristics into three states: hyperpolarizing (Pre_h_), depolarizing (Pre_d_) and a mixture of hyperpolarizing and depolarizing (Pre_m_) activities [35]. This information is then compared to the γ_j_(t) associated with the tonic firing patterns in the HMM to evaluate the correlation between the multiple model states with the intracellular dynamics.

Two types of spiking activities can be observed from the data set: tonic firing and chronic seizure activity. To evaluate the statistical robustness of the HMM, the sensitivity (TP) and specificity (TN) measures were determined. TP is defined by the percentage of correct chronic state detection within 30 s after the start of the chronic events, denoted as electrographical chronic seizure onset time (EcSOT) [44]. The percentage of correctly identified non-seizure/interictal activities at least 30 s prior to EcSOT is denoted as the TN. The ability of the HMM to detect chronic seizure onset early enough was determined. The time delay (*ΔT*) is defined as the time difference between the EcSOT and the approximated chronic seizure onset time (AcSOT) for each HMM topology. The AcSOT is defined as the time instance at which *γ*_*i*_*(t) *first identified the chronic seizure state within a detection horizon of 30 s around the EcSOT such that the EcSOT is located in the middle of a 1 min time window. Here, a positive *ΔT *would imply early chronic seizure detection while a negative *ΔT *would mean that the detection happened after the chronic seizure onset. Finally, the optimality index measure (*O*) [32], used to evaluate the overall performance of the HMM, is defined as:

Here, the value *d *is the chronic seizure duration for each SLE. A larger *O *indicates a better overall performance for a given detector. These measures (TP, TN, *ΔT *and *O*) from the HMMs were also compared with other supervised wavelet artificial neural network (WANN) [6] built on identical features. Figure 2 provides a graphical illustration on the overall training and evaluation strategies.

## Results

Comparison of the performance of HMM_opt7D _and HMM_opt14D _was done using a five-fold cross-validation technique. The model parameters of the HMMs were identified such that the best statistics for seizure detection in terms of optimality index (*O*) can be produced in the validation data set.

**Figure 3:**
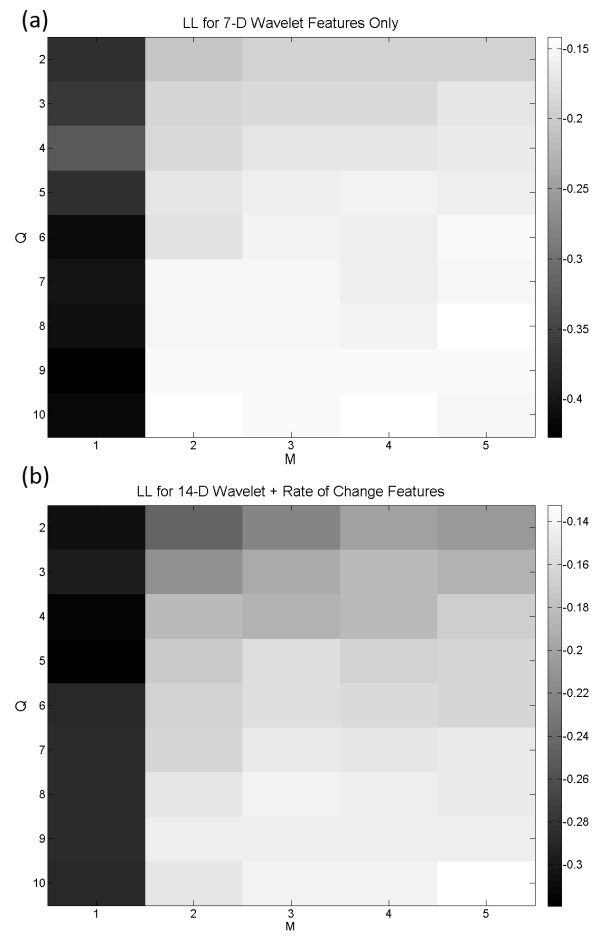
**Log-likelihoods of HMMs**. (a) The LL for the HMM using wavelet coefficient features is shown. The LL increases as Q and M goes up. (b) The LL for the HMM using both wavelet and rate of change features. For any particular Q and M combination, the LL in this model is always larger than Figure 3a.

After the unsupervised training and before the assignment of electrophysiological states to the model states, the LL for different combinations of Q and M values were compared to give the initial estimate on the goodness of fit between the model and the recorded data. Not surprisingly, the LL on the training data would increase and gradually reach a plateau for large Q and M values. However, using a large number of states and clusters would cause over-fitting due to the curse of dimensionality, hence severely jeopardizing the generalization ability of the detector. It was also not surprising that at identical Q and M combination, the LL for the 14-D HMM using both *c *and *Δc *was always larger than the LL for the 7-D HMM using *c *only (see Figure 3a and 3b). This implies that the feature space containing *Δc *may more accurately represent the underlying dynamics of SLEs.

**Figure 4:**
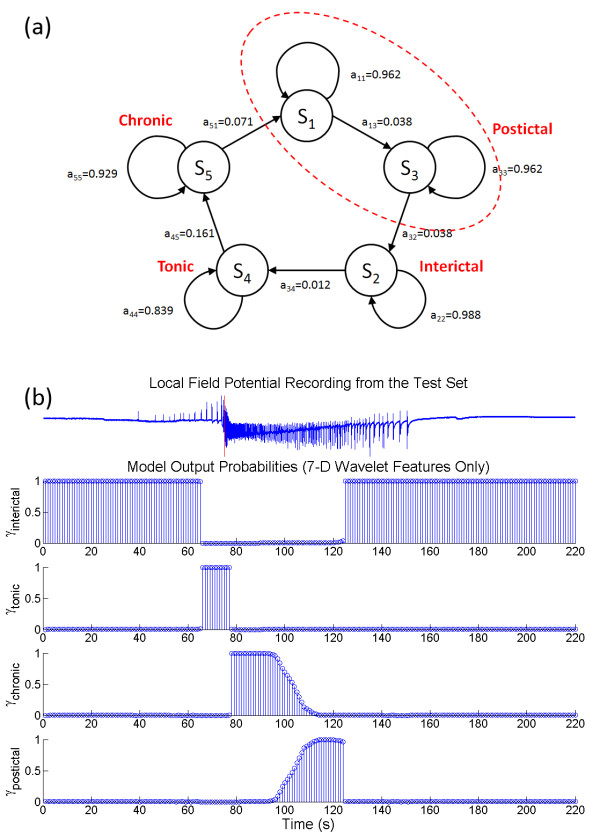
**The transition diagram and the posterior probabilities for HMM_opt7D _using the original feature space**. (a) A unidirectional sequence of state progress was found for the HMM_opt7D_. Even though the validation result indicated that a five state model should be used, *S*_*1 *_and *S*_*5 *_can be consolidated to represent the postictal events after expert guided state assignment. (b) The mapping of HMM_opt7D _output to the electrophysiological data is given. The marginal posterior probabilities illustrate that the HMM_opt7D _is able to detect the non-ictal, tonic firing, chronic seizures and postictal activities.

**Figure 5:**
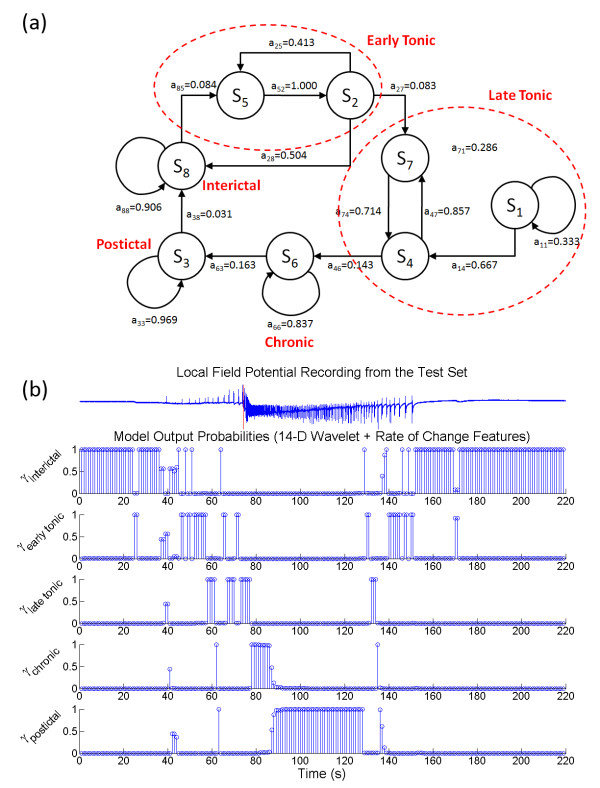
**The transition diagram and the posterior probabilities for HMM_opt14D _using the original feature space**. (a) The state transition diagram for HMM_opt14D _is given along with a sample LFP recording. For an eight state model, some of the states represent the same electrophysiological dynamics and hence can be consolidated. The HMM states *S*_*1*_, *S*_*4 *_and *S*_*7 *_represent the late tonic activities. They are interconnected and are always followed by the chronic activity and preceded by early tonic firing states *S*_*2 *_and *S*_*5*_. The early tonic activity of this HMM is also allowed to return to interictal state without generating seizures. (b) The mapping of HMM_opt14D _to the electrophysiological events is provided. Typically, this model is able to differentiate early and late tonic firing. For this particular example, the model output switches between interictal and early tonic state until about 46 s.

After assessing each HMM using the validation data, the topologies of the HMM_opt7D _and HMM_opt14D _were obtained with the expert-guided state assignment. For HMM with wavelet features (*c*) only, the maximum sensitivity of 68.4 ± 21.1% and specificity of 85.8 ± 13.7% on the validation set was obtained for the HMM topology of Q = 5 and M = 4. The *a*_*ij *_of the HMM_opt7D _also exhibited a unidirectional sequence of state transitions (as shown in Figure 4a). The state *S*_*5 *_was assigned as the chronic seizure state, S_5 _was preceded by *S*_*4 *_as the tonic firing state and *S*_*2 *_as the interictal state. The *S*_*1 *_and *S*_*3 *_can then be consolidated to represent the postictal activity. An illustration of the LFP in the test set along with the posterior probabilities of the model states is shown in Figure 4b. The HMM_opt14D _was found to contain Q = 8 and M = 3, with the state transition diagram shown in Figure 5a. The application of HMM_opt14D _on the validation set gave the maximum sensitivity of 80.1 ± 15.7% and maximum specificity of 95.3 ± 4.4%. The state *S*_*6 *_can be assigned as chronic seizure, *S*_*3 *_and *S*_*8 *_can be assigned as the postictal and interictal respectively. The three interconnecting states of *S*_*1*_, *S*_*4*_, and *S*_*7 *_before chronic state *S*_*6 *_suggested that they can be assigned as the late tonic firing activities. The state transition followed a somewhat unidirectional evolution except for the interconnecting states of the early tonic state (*S*_*2 *_and *S*_*5*_) back to the interictal state (*S*_*8*_), which appeared to suggest the possibility of having seizure permissive tonic firing not leading to chronic seizures. Figure 5b gives a graphical illustration of the LFP along with the posterior probabilities of the model states using HMM_opt14D_.

Next, the HMM approach was also compared against the supervised approach using identical wavelet coefficient feature set, in the form of a fully-supervised wavelet artificial neural network (WANN) seizure detection method designed by our group [6,7]. The training data for the WANN was created by first identifying the EcSOT, the data prior to the EcSOT was separated into 30 s intervals based on the assumption that there may be distinct changes in LFP leading to the EcSOT. Based on one-way ANOVA statistical analysis, the WANN and HMMopt7D did not show significant difference in their optimality index (0.756 ± 0.059 and 0.665 ± 0.260, respectively).

When the wavelet rate of change information was included in the feature space, the HMM_opt14D _gave the best overall performance out of the three approaches, with *O *= 0.915 ± 0.302 (p < 0.005). Table 3 summarizes the result of the chronic seizure detection for these three models.

**Table 3:** Performance measure for supervised and unsupervised seizure detection approach.

	WANN	**HMM**_**opt7D**_	**HMM**_**opt14D**_
Sensitivity (TP)	73.1 ± 3.7%	69.8 ± 20.3%	86.7 ± 27.2%
Specificity (TN)	91.7 ± 4.4%	88.1 ± 20.9%	98.6 ± 7.7%
Detection delay (ΔT)	3.95 ± 3.38 s	8.30 ± 15.33 s	-0.68 ± 10.07 s
Optimality index (O)	0.756 ± 0.059	0.665 ± 0.260	0.915 ± 0.302

Using identical wavelet coefficient features, the performance of the unsupervised HMM seizure detector (HMM_opt7D_) did not show any statistically significant improvement over the supervised approach (WANN). However, when the rate of change of wavelet information was included in the feature space, the optimality index of HMM_opt14D _was significantly better than both WANN and HMM_opt7D_.

**Figure 6:**
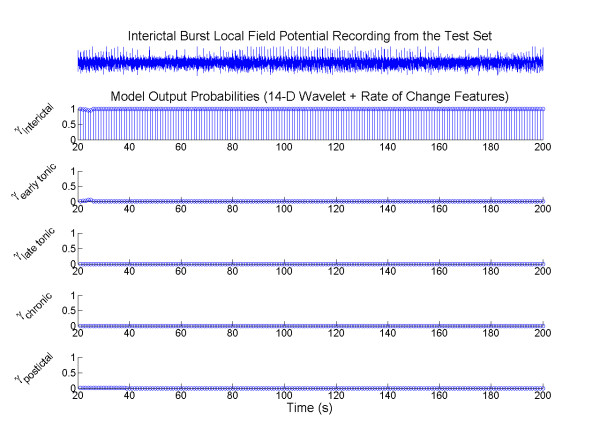
**State detection of interictal burst activities**. An illustrative example of applying the HMM to the interictal burst data. Even though bursting activity is exhibited, the HMM is able to accurately classify the LFP as interictal state when the wavelet coefficient and rate of change features are used.

The next question we asked was whether the state transitions of the HMM would hinder the performance of the state detection on seizure-free data as well as seizure permissive states that could translate back into interictal without generating seizures. Using 30 min of interictal bursts and non-seizure data that were not part of the training set, mean detection accuracies of 90.1% and 97.1% were achieved using HMM_opt7D _and HMM_opt14D _respectively. Since the state transition of HMM_opt7D _is strictly unidirectional (Figure 4a), any missed false detection of the interictal state would trigger a cascade of errors, resulting in significantly lower accuracy in interictal state detection. HMM_opt14D _on the other hand allowed possible reversal in state transition back to interictal. In Figure 6, a sample LFP recording along with the posterior probabilities of the HMM_opt14D _is shown, illustrating that the model was able to classify the IB activities as non-ictal/interictal.

**Figure 7:**
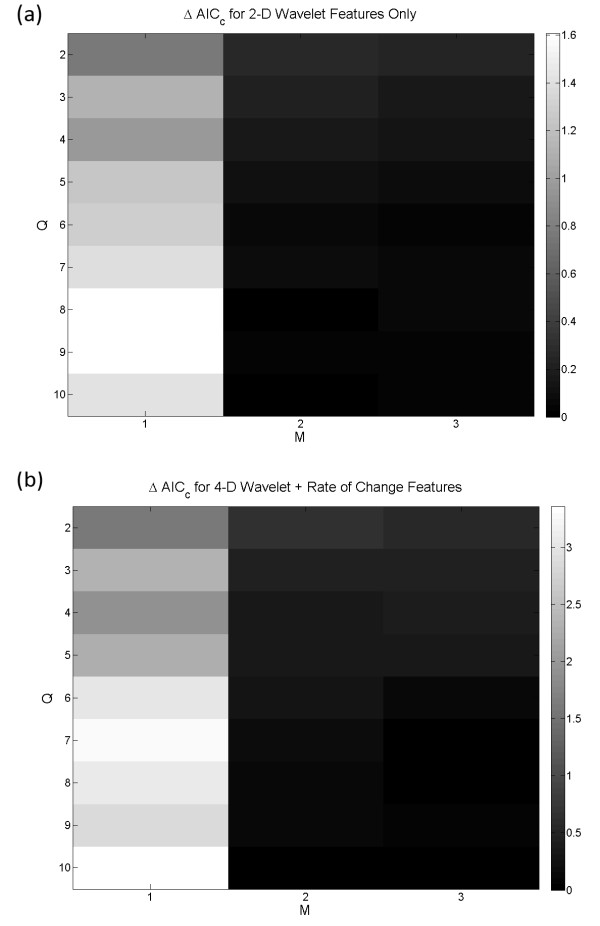
**The rescaled AICc values are plotted with respect to the number of HMM states and Gaussian mixtures**. (a) The simplest model with ΔAICc < 0.25 is at Q = 3 and M = 2. In this model, the reduced feature space consists of 2-D wavelet coefficients. (b) With the 4-D wavelet and rate of change features established using mRMR, the simplest model with ΔAICc < 0.25 is found at Q = 5, M = 3.

**Figure 8:**
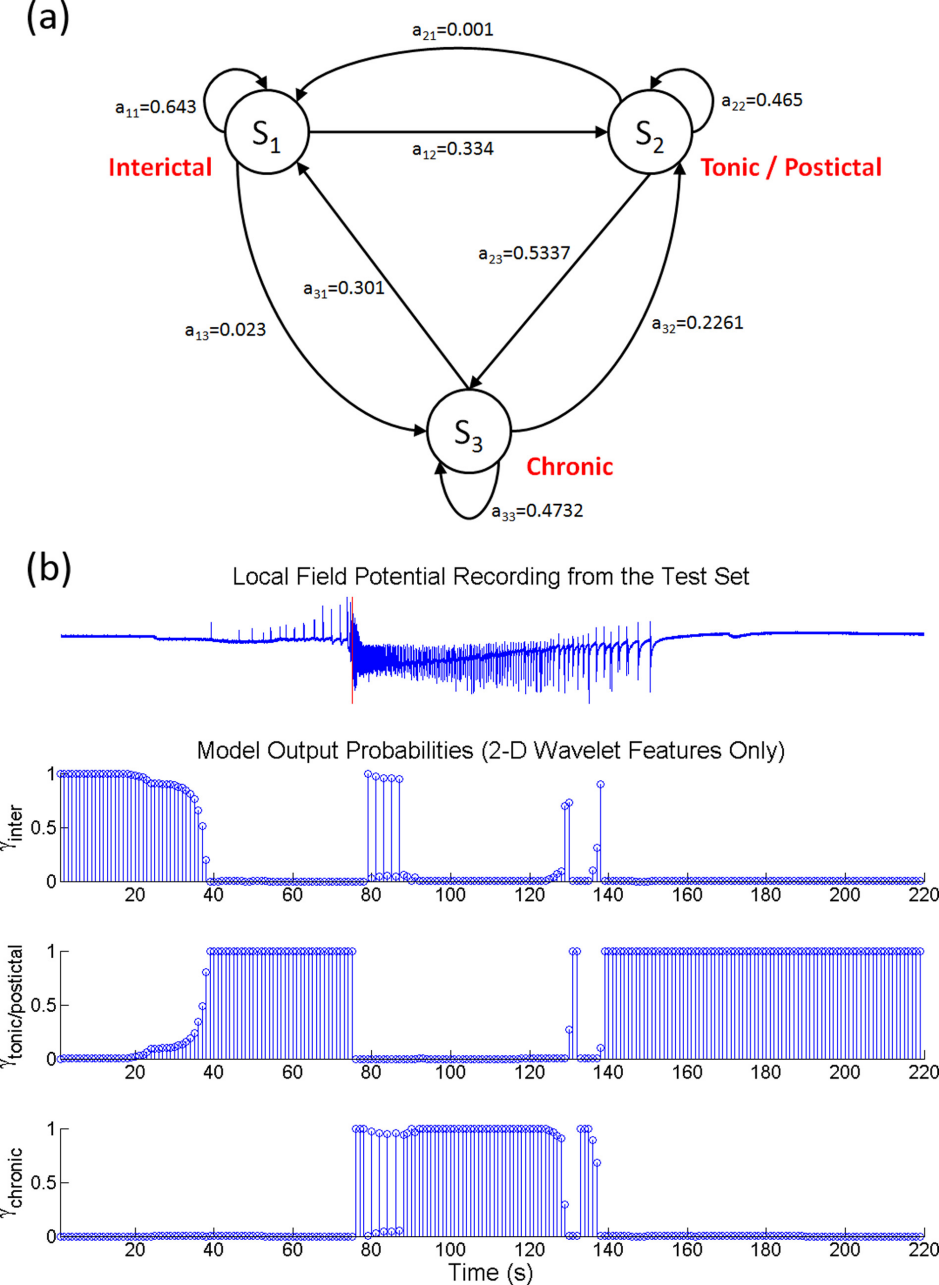
**The transition diagrams and the posterior probabilities of the reduced feature space after mRMR and AICc**. (a) A bidirectional sequence of state progress is found where the HMM is not able to distinguish between tonic firing and postictal activities. (b) The mapping of the HMM output to the experimental data is shown. The tonic firing phase prior to the chronic seizure and the postictal activity are represented by the same model state. Because the state transition probability from the tonic/postictal state is extremely small (0.001), it would take much longer for the interictal activity right after chronic seizure to be classified correctly.

**Figure 9:**
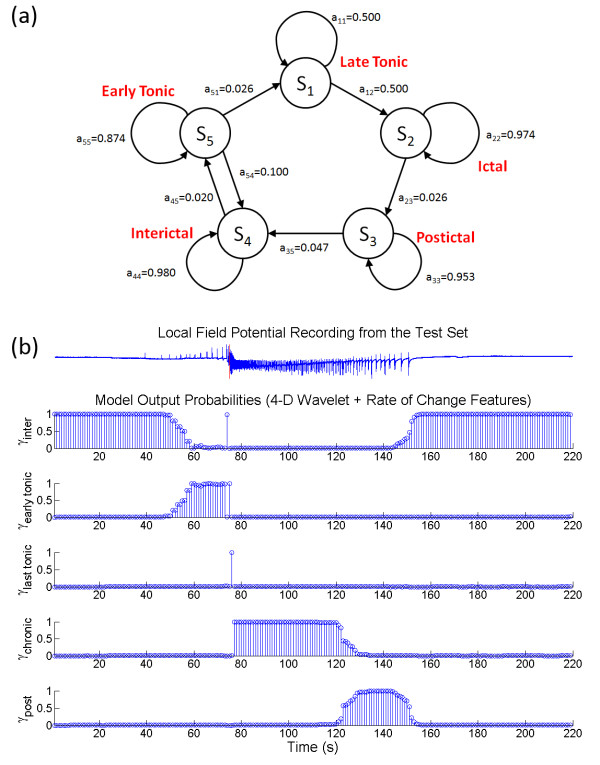
**The transition diagrams and the posterior probabilities of the reduced feature space after mRMR and AICc**. (a) The state transition diagram for HMM_AIC _is given. Similar to the HMM_opt14D_, the some early tonic activity permissive to seizures is allowed to return to the interictal state. Between the late tonic firing and postictal state, the transition follows a unidirectional progression. (b) The mapping of HMM_AIC _states to the electrophysiological LFP is shown. In this illustrative example, the marginal probabilities of the model states follow a progression from non-ictal, tonic firing to chronic seizures and postictal state, before returning to non-ictal. The late tonic firing state in this model is typically shorter than the model from Figure 5b because of the significantly smaller transition probability back to itself.

Another method for selecting the appropriate HMM topology was investigated by reducing the feature space dimension and the number of free parameters in the model. Minimum redundancy maximum relevance (mRMR) technique was used to select a subset of relevant wavelet features. It can alleviate the effect of over-fitting caused by the curse of dimensionality and improve the model's ability to generalize. In conjunction with the AICc, which helped balance the LL against the number of model parameters, suitable optimum labelled HMM_AIC _can be found. A summary of the mRMR analysis is shown in Table 4. The wavelet coefficients associated with the alpha (8 - 15 Hz) and beta (15 - 40 Hz) bands had the largest mutual information quotient (*V*_*F*_*/W*_*c *_= 0.497 and 0.436 respectively). The wavelet coefficients at these two frequency bands then constituted the reduced features space. For the feature space consisted of the wavelet coefficients (*c*) only, the simplest 2-D HMM topology with ΔAICc < 0.25 was found at Q = 3, M = 2 (Figure 7a). The state transition diagram of the corresponding HMM is shown in Figure 8a. It contained three bidirectionally connected states. When the posterior probabilities of this model were matched against the LFP after assigning the model states (Figure 8b), no distinction between the tonic firing and the postictal events was found. There were also cases in which the state transitions jumped from interictal to chronic seizure directly and back. ON average, the performance of this HMM was slightly better than the HMM_opt7D_, with TP = 80.9 ± 34.4%, TN = 94.8 ± 15.6%, *ΔT *= 3.79 ± 8.67 s and *O *= 0.813 ± 0.247, even though student T-test analysis did not reveal any statistically significant improvement. When the wavelet rate of change information (*Δc*) was added as feature, a 4-D reduced feature space was created. The simplest topology with ΔAICc < 0.25 for this feature set was found at Q = 5 and M = 3 (Figure 7b). Figure 9a shows the state transition diagram for this HMM (called HMM_AIC_). Similar to the HMM_opt14D_, the state progression was mainly unidirectional with a non-zero state transition probability from the early tonic state back to the interictal state. This suggests that it is possible to have seizure permissive early tonic activity that can be reverted back to the interictal state. The marginal posterior probability *γ*_*i*_*(t) *for each state *i *of HMM_AIC _is plotted against a sample test data in Figure 9b. Even though no significant improvement over HMM_opt14D _was revealed using the student T-test, the HMM_AIC _gives the best overall performance out of all the HMMs created in this study with TP = 95.7 ± 14.0%, TN = 98.9 ± 6.5%, *ΔT *= -2.03 ± 7.10 s and *O *= 0.995 ± 0.129. A summary of the performance measures for the HMM_AIC _using mRMR and ΔAICc is presented in Table 5.

**Table 4:** Feature selection using mRMR method.

Order	**V**_**F **_**/W**_**c**_	Frequency Range (Hz)
1	0.497	8 - 15
2	0.436	15 - 40
3	0.400	100 - 250
4	0.331	4 - 8
5	0.308	40 - 100
6	0.308	250 - 400
7	0.266	<4

The frequency bands associated with the two highest mutual information quotients (V_F_/W_c_) were used to create the reduced feature space. In our analysis of chronic seizures versus non-seizure activities, the alpha (8 - 15 Hz) and beta (15 - 40 Hz) activities were found to be the most relevant for seizure detection.

**Table 5:** A summary of HMM performance measures.

Features	Wavelet Coefficients Only		Wavelet Coefficients + Rate of Change	
	**Validation Set**	**mRMR and ΔAICc**	**Validation Set**	**mRMR and ΔAICc**
	**7-D (Q = 5, M = 4)**	**2-D (Q = 3, M = 2)**	**14-D (Q = 8, M = 3)**	**4-D (Q = 5, M = 3)**

Sensitivity (TP)	69.8 ± 20.3%	80.9 ± 34.4%	86.7 ± 27.2%	95.7 ± 14.0%
Specificity (TN)	88.1 ± 20.9%	94.8 ± 15.6%	98.6 ± 7.7%	98.9 ± 6.5%
Detection delay (ΔT)	8.30 ± 15.33 s	3.79 ± 8.67 s	-0.68 ± 10.07 s	-2.03 ± 7.10 s
Optimality index (O)	0.665 ± 0.260	0.813 ± 0.247	0.915 ± 0.302	0.995 ± 0.129

The optimal HMM model topologies were determined using two methods. The first method involved the use of validation data set consisting of 10 SLEs to find the HMM topology that gave the highest optimality index. The second method used the mRMR feature selection method and AICc to reduce the feature space dimension and the complexity of the HMM. The HMM obtained using mRMR and AICc has the highest optimality index.

**Figure 10:**
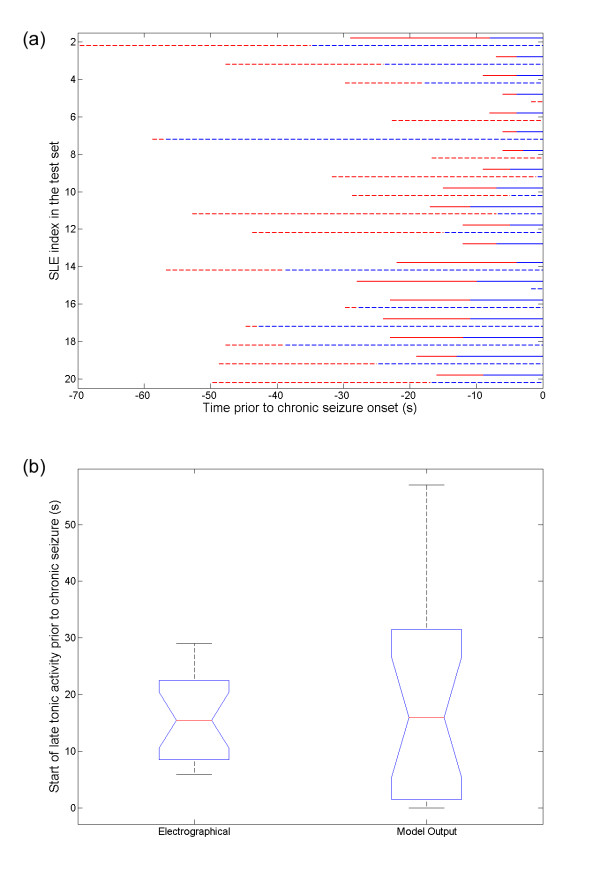
**The relationship between the HMM identified late tonic states and the intracellular modes**. (a) The temporal relationship between the intracellular modes before chronic seizures and the HMM late tonic firing states for each SLE in the test set is shown. The intracellular modes are shown in solid color (red for pre_m _and blue for pre_d_). The identified states by the HMM are shown as dashed lines (red for early tonic and blue for late tonic states). (b) The boxplot summarizes the relationship between the start of the pre_d _intracellular activity and the onset of the late tonic firing model state. The HMM was able to identify 85.9% of the pre_d _activity as the late tonic firing state. The mean starting times for the electrographical pre_d _activity and the model late tonic state were 16.25 s and 16.77 s prior to chronic seizure onset, respectively. Using pair-wise T-test analysis, no statistically significant difference was found between the two (p > 0.49).

Finally, we evaluated the correlations between the model tonic firing state from the HMM_AIC _with the phasic inhibition or excitation in the intracellular activities [35]. It has been suggested that the intracellular whole-cell recordings exhibited a switch from a dominant phasic inhibition (pre_h_) to a dominant phasic excitation (pre_d_) mode in the state transition leading to the chronic seizure onset [35]. An intermediate state (pre_m_) was also reported to compose of a mixture of pre_h _and pre_d _mode. None of the HMM created in this study was able to detect early pre_h _mode using the LFP data. In the HMM_opt14D_, the states *S*_*2 *_and *S*_*5 *_(Figure 5a) can be considered as a combination of late pre_h _and pre_m_. Figure 10a summarizes the temporal relationship between the HMM-identified early and late tonic firing activities in the LFP with the identified pre_m _and pre_d _modes in the whole-cell recording. Most of the late tonic firing activities identified by the model started earlier than the pre_d _intracellular activities. Out of the 20 test cases, 85.9% of the pre_d _activity was identified as the late tonic firing phase. A paired T-test did not indicate a statistically significant difference between the start of the whole-cell pre_d _activity and the onset of the late tonic HMM state (p > 0.49), as illustrated in Figure 10b.

## Discussion

The administration of a therapeutic intervention, such as electrical stimulation, may be effective in preventing seizures before and during the early stages of the seizure onset [45]. The success of a real-time closed-loop seizure prevention method depends on the time available between the early seizure detection and the manifestation of ictal onset. Many early seizure detection algorithms have been proposed [9,15,23,32,46-50]. The application of HMM for seizure dynamics detection was inspired by the relatively poor objective criteria for identifying the precise period of preictal interval in supervised learning. Here, an unsupervised training strategy with expert guided state assignment of HMM is proposed where the best rule to represent the wavelet features of seizure progression can be identified. One of the major criteria for selecting an appropriate seizure detection algorithm for a real-time seizure intervention system is the available time for the detection of the impending ictal onset. In this paper, HMMs were created utilizing different feature vectors to characterize the dynamics of SLEs. A motivation behind this work is to determine whether an unsupervised method can produce an accurate seizure detector with a high optimality index. Furthermore, feature selection based on the mRMR criteria and topology selection based on ΔAICc were implemented to evaluate the ability of the HMM to detect multi-stage dynamics leading to chronic seizure onset by measuring the sensitivity, specificity, detection delay and the optimality index.

The unsupervised learning strategy of the HMM involves the estimation of the model parameters through the maximization of the LL function. The training data consists of the wavelet-based features from 20 SLEs. Unlike other recent approaches that assumed a fixed number of states (such as interictal, preictal and ictal) in the model [28], the total number of hidden states in the HMM in this study was allowed to vary in an unsupervised manner. It is logical to expect that if more states are allowed, the better the model would match the observations. However, increasing the possible number of hidden states would lead to over-fitting, hence reducing its ability to generalize. To alleviate this problem, we need to find out the optimal HMM topology defined by the number of states (Q) and the number clusters in the feature space (M) that would be able to generalize well.

The first method we tried was to set aside a portion of the data for validation purpose. The objective was to get the Q and M combination that would give the highest optimality index in the validation set. The optimal HMM (HMM_opt7D_) created using only wavelet coefficient features had five states (Q = 5) with each feature space modeled by four clusters (M = 4). If the rate of change of the wavelet coefficients was also included in the feature space, the optimal HMM (HMM_opt14D_) would consist of eight states (Q = 8) with the feature space modeled using three clusters (M = 3). The HMM_opt14D _is superior to the HMM_opt7D _not only because it has a higher optimality index (0.915 ± 0.129, compared to 0.665 ± 0.260), but also because it is more robust in identifying multiple distinct dynamics between non-ictal and ictal events. The detection of early and late tonic firing activities prior to the chronic onset *in vitro *also became possible. While the training process of the HMM was not constrained to follow a unidirectional state transition, the state transitions leading to the ictal onset often possessed some unidirectional progress. Once the HMM_opt7D _output moved away from the interictal state, it must go through the whole seizure progression before returning to interictal. This would then increase the number of false detection in HMM_opt7D_, as indicated by the results shown in Table 3. On the other hand, some early tonic firing state in the HMM_opt14D _was allowed to revert back to interictal directly. However, once it had advanced past the early tonic state, it could not revert back to non-ictal activities. The HMM_opt14D _was successfully tested on non-seizure or interictal burst data, demonstrating a mean accuracy of over 97%. Next, the unsupervised HMM and supervised WANN approaches [6] were compared. The two methods differ in the way that the parameters are obtained. The parameters of the HMM were updated in an iterative manner until no significant improvement in LL was achieved. The supervised WANN approach, on the other hand, required that the human user separated the data into different training groups. The WANN parameters were updated iteratively based on the partial derivative of error with respect to the weights. By definition, the performance of any supervised learning algorithm can be no better than the initial separation of seizure states by the human expert's "Gold Standard". Since the human user has access to the entire observation before marking the chronic onsets, retrospective bias exists in any supervised learning algorithm [28]. This retrospective bias can be eliminated with an unsupervised algorithm such as HMM. The HMM implementation is superior to the WANN because it can distinguish between the early and late tonic firing without having to define them before training.

The second method to reduce the complexity of the HMM based on mRMR feature selection criteria and ΔAICc topology selection was evaluated. The mRMR analysis showed that activity in the alpha (8 - 15 Hz) and beta (15 - 40 Hz) range has the largest mutual information quotient (*V*_*F*_*/W*_*c*_) of 0.497 and 0.436 respectively. This result is consistent with the existing literature showing that the alpha and beta frequency bands exhibit considerable difference in the signal complexity between healthy subjects, epileptic subjects during a seizure-free interval, and during seizure [8]. Two new feature spaces were constructed: One consisted of 2-D wavelet coefficients and one consisted of 4-D wavelet coefficient with rate of change information. Using these new feature spaces, HMMs with different topologies were created using unsupervised learning. Since having large number of Q and M often lead to the curse of dimensionality resulting in over-fitting, AICc was used to select the appropriate model topology by balancing the goodness of fit with the number of parameters used. We found that many Q and M combinations were able to achieve similar levels of AICc; the optimal HMM was selected as the model with the smallest number of parameters while maintaining a difference of <0.25 from the minimum AICc. The HMM of 2-D reduced features consists of three states (Q = 3) with two clusters (M = 2). One of these three states reflects the dynamics of both tonic firing and postictal activities (Figure 8a). Because of the small transition probability (*a*_*ij *_= 0.001) from the tonic/postictal back to interictal state, it typically would take a much longer time for the model to return to the proper interictal state after chronic seizure. This has been the source for most of the misclassification in the non-ictal activity using this model. The HMM model of 4-D reduced features consists of five states (Q = 5) with three clusters (M = 3). Again, by including the wavelet rate of change information, this model gave the best overall performance with *O *= 0.995 ± 0.129, even though the use of AICc does not warrant an improvement in seizure detection performance. The AICc simply offers a much simpler alternative to selecting the optimal HMM topology. We were able to more easily and more quickly create a HMM seizure detector with fewer number of model states and feature clusters with similar performance level.

The interpretation of early and late tonic firing state detected using HMM_AIC _in terms of the underlying intracellular whole-cell measurement was investigated. When the model posterior probabilities were plotted against the corresponding intracellular whole-cell recording, we found a significant overlap (>85%) between the late tonic state and the intracellular pre_d _activity. It was also observed that the early hyperpolarizing activity (pre_h_) could not be distinguished from the interictal activities.

The application of HMM is based on the assumption that state transitions in seizure generation follow first-order Markov processes. Depending on the sampling period of the signal and the available history of HMM output, it is likely that neurodynamic would depend on more than one previous sample in time. This work can be extended to incorporate additional states in the past for the estimation of state transitions, which can be achieved through modification of the conditional state transition probability equation such as the hidden semi-Markov model [51] where state transition depends on the lapsed time since entering the current state. This model is also appropriate because the underlying process of seizure generation may not have a geometrically distributed duration. Another possible improvement is to include other types of features such as multi-site coherence in the feature space, since the manifestation of epilepsy typically involved progressive global entrainment. Preliminary analysis on the analysis of clinical seizure data from intracranial EEG measurement using HMM also showed promising results [52].

## Conclusions

Based on the analysis of optimality index, the implementation of hidden Markov model as a seizure dynamics detector offers significant improvement over existing approaches based on human visual classification and supervised connectionist perspectives. The subjectivity involved in partitioning the observed data into target states prior to training is eliminated. This model is able to estimate the parameters needed to best fit the observed data depending on its specified topology. Once the unsupervised training is completed, the assignment of the model state to the electrophysiology data is guided by a human expert. To alleviate the curse of dimensionality, feature selection based on minimum redundancy maximum relevance and topology selection based on Akaike information criteria are implemented without jeopardizing the sensitivity, specificity and early detection time of the final model. A five-state hidden Markov model was created, capable of detecting chronic seizures with 95.7% sensitivity and 98.9% specificity. It was also able to detect early and late tonic firing activities that correlate with the intracellular whole-cell dynamics prior to chronic seizure onset. Such a model would potentially allow the researchers to decipher possible sequence of distinct dynamic modes leading to seizure onsets.

## Competing interests

The authors declare that they have no competing interests.

## Authors' contributions

AWLC and SOT carried out the data analysis and drafted the manuscript. PLC supervised and MD and MC performed the data acquisition. AWLC and BLB participated in the design of the study. All authors read and approved the current manuscript.
